# Feasibility, Safety, and Efficacy of Pressurized Intraperitoneal Aerosol Chemotherapy (PIPAC) for Peritoneal Metastasis: A Registry Study

**DOI:** 10.1155/2018/2743985

**Published:** 2018-10-24

**Authors:** Florian Kurtz, Florian Struller, Philipp Horvath, Wiebke Solass, Hans Bösmüller, Alfred Königsrainer, Marc A. Reymond

**Affiliations:** ^1^Dept. of General Surgery, Karls-Eberhard University Tübingen, Germany; ^2^Institute of Pathology, Karls-Eberhard University Tübingen, Germany; ^3^National Center for Pleura and Peritoneum, Comprehensive Cancer Center South-Western Germany, Tübingen, Stuttgart, Germany

## Abstract

**Introduction:**

Pressurized intraperitoneal aerosol chemotherapy (PIPAC) is a novel drug delivery system with superior pharmacological properties for treating peritoneal metastasis (PM). Safety and efficacy results of PIPAC with cisplatin/doxorubicin or oxaliplatin from a registry cohort are presented.

**Methods:**

IRB-approved registry study. Retrospective analysis. No predefined inclusion criteria, individual therapeutic recommendation by the interdisciplinary tumor board. Safety assessment with CTCAE 4.0. Histological assessment of tumor response by an independent pathologist using the 4-tied peritoneal regression grading system (PRGS). Mean PRGS and ascites volume were assessed at each PIPAC.

**Results:**

A total of 142 PIPAC procedures were scheduled in 71 consecutive patients with PM from gastric (*n* = 26), colorectal (*n* = 17), hepatobiliary/pancreatic (*n* = 9), ovarian (*n* = 6), appendiceal (*n* = 5) origin, pseudomyxoma peritonei (*n* = 4), and other tumors (*n* = 3). Mean age was 58 ± 13 years. Patients were heavily pretreated. Mean PCI was 19 ± 13. Laparoscopic nonaccess rate was 11/142 procedures (7.7%). Mean number of PIPAC/patient was 2. All patients were eligible for safety analysis. There was no procedure-related mortality. There were 2.8% intraoperative and 4.9% postoperative complications. 39 patients underwent more than one PIPAC and were eligible for efficacy analysis, and PRGS could be assessed in 36 of them. In 24 patients (67%), PRGS improved or remained unchanged at PIPAC#2, reflecting tumor regression or stable disease. Ascites was present in 24 patients and diminished significantly under therapy. Median survival was 11.8 months (95% CI: 7.45–16.2 months) from PIPAC#1.

**Conclusion:**

PIPAC is feasible, safe, and well-tolerated and can induce histological regression in a significant proportion of pretreated PM patients. This trial is registered with NCT03210298.

## 1. Introduction

In spite of recent progress in systemic palliative chemotherapy, prognosis of peritoneal metastasis remains dismal [1]. The cytotoxic effect of chemotherapy might be improved by the use of intraperitoneal drug delivery, when malignant disease is confined to the peritoneal cavity. Improved cytotoxicity is based on the theoretical potential for increased exposure of the tumor to antineoplastic agents [2].

However, two pharmacokinetic problems appear to limit the effectiveness of intraperitoneal chemotherapy: poor tumor penetration by the drug and incomplete irrigation of serosal surfaces by the drug-containing solution [3, 4] Moreover, intraperitoneal chemotherapy is hampered by dose-limiting local toxicity, so that its clinical use remains fairly limited [5]. Thus, there is a considerable research effort for developing next-generation drug delivery systems for intraperitoneal chemotherapy that maximizes local efficacy while limiting systemic side effects [6].

One of these new intraperitoneal cytotoxic drug delivery systems is pressurized intraperitoneal aerosol chemotherapy (PIPAC), which is gaining rapid clinical acceptance in the palliative therapy of peritoneal metastasis worldwide. The rationale of PIPAC is based on the use of a pressurized therapeutic aerosol [7] with superior pharmacological properties, in particular, a deeper drug tissue penetration [8, 9], a higher drug tissue concentration [10], and a more homogeneous distribution than with liquid chemotherapy [11, 12]. Increased drug penetration under pressure had been previously demonstrated in other animal models [13, 14]. Moreover, hyperpressure obtained by gas administration during laparoscopy decreases the venous blood outflow from the abdomen, which might result in an increased time of drug tissue contact [15]. PIPAC allows a significant dose reduction and therefore largely prevents systemic organ toxicity [16, 17]. Promising results have been published in peritoneal metastasis of gastric [18, 19], ovarian [20, 21], colorectal [22], pancreatic [23, 24], and hepatobiliary [25] origins. A recent systemic review of the literature concluded that PIPAC is feasible, safe, and well-tolerated and that preliminary good response rates call for a prospective analysis of oncological efficacy [26].

Meanwhile, at least 15 prospective clinical trials are ongoing evaluating oncological efficacy of PIPAC in multiple indications with various drugs, including cisplatin, oxaliplatin, doxorubicin, and nab-paclitaxel [27]. However, these studies only cover selected groups of patients so that registry data are needed for highlighting PIPAC results in the other patients and in rare indications. In the present paper, we report about our experience gained with PIPAC since July 2016 at a tertiary cancer center offering all other therapeutic options for peritoneal metastasis, including in particular palliative systemic chemotherapy, intraperitoneal chemotherapy, intraperitoneal virotherapy [28], cytoreductive surgery, and hyperthermic intraperitoneal chemotherapy (HIPEC).

Here, we demonstrate that PIPAC is feasible in a large number of patients with peritoneal metastasis in the salvage situation, that PIPAC is safe, and that encouraging survival figures and significant ascites reduction has been observed.

## 2. Patients and Methods

### 2.1. Study Design

This is a registry study on a cohort of consecutive patients, starting with the first patient treated with PIPAC in our institution. No patient was excluded. Data were entered prospectively into a patient registry, and analysis was retrospective. No primary/secondary endpoint was predefined.

### 2.2. Ethical and Regulatory Framework

The international PIPAC patient registry was approved by the Ethics Committee, Ruhr-University Bochum, on Jan 11, 2016 (reference 15-5280), and by the data protection officer of the State of North Rhine-Westphalia, Germany. Each patient gave his/her written informed consent both for the PIPAC procedure and for data storage management and analysis. The registry is hosted by an independent quality control organization (AnInstitut für Qualitätssicherung in der Operativen Medizin gGmbH, Otto-von-Guericke Universität Magdeburg) [29]. Although cisplatin, doxorubicin, and/or oxaliplatin is routinely used in clinical practice worldwide for locoregional therapy in peritoneal disease and has been the object of multiple randomized controlled trials [30–37], none of these drugs is currently approved for intraperitoneal delivery. Therefore, PIPAC was applied “off-label.”

### 2.3. Patient Selection

Each patient was presented in the interdisciplinary tumor board (ZGO) of the Comprehensive Cancer Center, University Hospital Tübingen, Germany. PIPAC therapy was recommended on an individual basis; no inclusion or exclusion criteria were predefined. Patients with extraperitoneal metastases (with the exception of isolated pleural effusion) were not treated. Complete or partial bowel obstruction, presence of gastric discharge tube, or very poor general condition (Karnofsky < 50%) was considered a palliative situation, and the patients were referred to a palliative care unit. If the tumor board recommended cytoreductive surgery and HIPEC, then the patient was not considered for a PIPAC therapy.

### 2.4. Technique of PIPAC

After the creation of a standard CO_2_ pneumoperitoneum, two access trocars (Kii®, Applied Medical, Düsseldorf, Germany) were inserted through the abdominal wall. A staging laparoscopy with the determination of the peritoneal cancer index (PCI) was followed by multiple peritoneal biopsies in all abdominal quadrants. Tightness of the abdomen was verified. A disposable nebulizer (Capnopen®, Capnomed GmbH, Villingendorf, Germany) was introduced under videoscopic control into the abdomen through one of the trocars and connected with a high-pressure injector through a dedicated high-pressure line. Patients with peritoneal metastasis of colorectal or, by analogy, of appendicular origin were treated with oxaliplatin 92 mg/m^2^ BSA diluted into 150 ml Glc 5%. All other patients were treated sequentially by doxorubicin 1.5 mg/m^2^ BSA diluted into 50 ml 0.9% saline, then with cisplatin 15 mg/m^2^ BSA diluted into 150 ml 0.9% saline. The cytotoxic solutions were nebulized into the expanded peritoneal cavity under an upstream pressure of 20 bar. Then, the therapeutic capnoperitoneum was maintained in a steady state at a pressure of 12 mmHg at a temperature of 37°C for 30 min. Thereafter, the capnoperitoneum was deflated through a closed aerosol waste system (CAWS) and the procedure was terminated.

### 2.5. Occupational Health Safety

The following safety measures were taken to exclude any exposure of the personnel [38]: first, the tightness of the abdomen was documented via zero-flow CO_2_. Second, the procedure was performed in an operating room equipped with a laminar airflow. Third, the chemotherapy injections were remote-controlled, and no personnel remained in the operating room during the application.

### 2.6. PIPAC Cycles

PIPAC was repeated at 6-week intervals; in the case of major or complete histological regression, this interval was extended to 3 months. In the cases of combined treatment (systemic and intraperitoneal chemotherapy), a minimum delay of two weeks (bevacizumab: 4 weeks) between the last application of systemic chemotherapy and PIPAC was observed. Systemic chemotherapy could be started immediately (in practice 1 week) after PIPAC application.

### 2.7. Efficacy Assessment

Histological regression was assessed by an independent pathologist (Department of Pathology, University of Tübingen, Germany) by grading tumor biopsies taken during each PIPAC. Patients eligible for histological tumor response assessment had at least 2 PIPAC cycles. Histopathological tumor regression was graded according to the 4-tied peritoneal regression grading system (PRGS) [39]. Ascites was removed at the beginning of each PIPAC procedure, and the volume was measured. Analysis has been performed on 25 patients who presented with ≥300 ml ascites at PIPAC 1. All 25 patients were included in the analysis, independently of the number of PIPAC cycles received. PCI was not used as response criteria, due to the large subjectivity in the macroscopic judgement of lesions (tumor vs. scar).

### 2.8. Safety Assessment

Adverse events were graded according to the Common Terminology Criteria for Adverse Events (CTCAE) version 4.0 [40]. Surgical complications were graded according to the Dindo-Clavien classification [41].

### 2.9. Follow-Up

The mean follow-up was 10.4 ± 4.2 months. The closing date was October 17, 2017. A staging CT-scan was recommended every 3 months. Patients and/or general practitioners were contacted by phone and/or email.

### 2.10. Statistical Analysis

All *p* values are two-tailed, and a *p* value of less than 0.05 was considered statistically significant. Values are given as means or medians, where appropriate. We performed a multivariable logistic regression model (Cox) with survival as the dependent variable and PCI, Karnofsky index, PRGS, time point of peritoneal metastasis (synchronous vs. metachronous), number of previous chemotherapy lines, and presence of ascites (yes/no) as independent variables. Survival was modelled in a Kaplan-Meier survival curve. We used SPSS 24 for Windows (SPSS Inc., Chicago, IL, USA) for statistical analysis and SigmaPlot 13 (Systat Software Inc., San José, CA, USA) for creating graphs.

## 3. Results

### 3.1. Patients and Procedures

A total of 142 PIPAC procedures were scheduled in 71 patients with peritoneal metastasis and gastric cancer (*n* = 26), colorectal cancer (*n* = 17), hepatobiliary/pancreatic cancer (*n* = 9), ovarian cancer (*n* = 6), appendiceal cancer (*n* = 5), pseudomyxoma peritonei (*n* = 4), and other tumors (*n* = 3). The mean age of this cohort was 58 ± 13 years. The Karnofsky index was 80.3 ± 14.7. Ascites (300 ml and more) and pleural effusion were present in 24 and three patients, respectively. 42/71 patients received combined systemic chemotherapy and PIPAC. Patients' characteristics including the number of previous chemotherapy lines are shown in Table 1.

### 3.2. Feasibility

**Figure 1:**
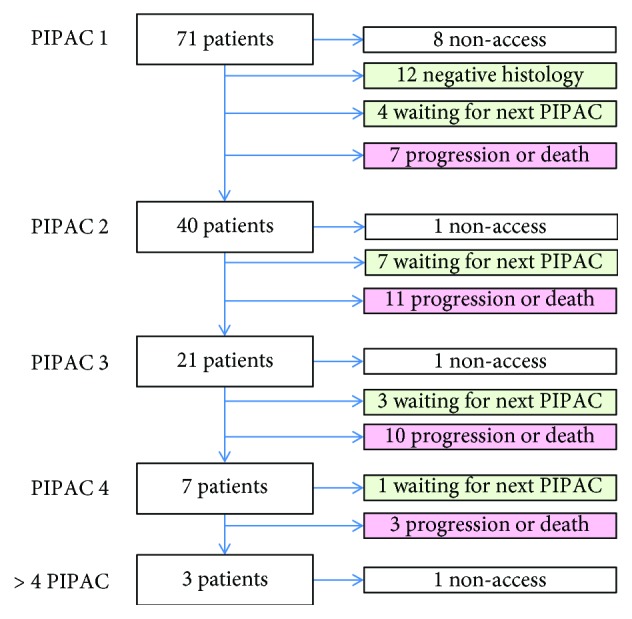
Patient flowchart.

The patient flowchart is detailed in Figure 1. The laparoscopic nonaccess rate was 11/142 procedures (7.7%; eight primary, three secondary nonaccess). Thus, 24, 19, 13, six, and one patients underwent successfully one, two, three, four, and six PIPAC, respectively, totalizing 131 successful procedures. The mean number of PIPACs administered was two (minimum one, maximum six). The mean operating time was 103 ± 30.7 min. The procedures were performed by six different surgeons, including two residents.

### 3.3. Safety

All 71 patients were eligible for safety analysis. Complications and side effects are detailed in Table 2.

- Intraoperative complications: there were four (2.8% procedures) intraoperative complications: one lung aspiration ad induction of narcosis, one bowel laceration in a patient with tumoral adherence to the abdominal wall, one bowel puncture with the Veres needle, and one bleeding requiring laparotomy. All surgical complications were repaired intraoperatively, and the patients recovered uneventfully. In all four patients, application of PIPAC was postponed to a later point of time
- Postoperative complications: there were seven postoperative complications (4.9% procedures): two patients developed abdominal wall infiltration because of localized leakage of the toxic aerosol, one patient under systemic chemotherapy (cisplatin/gemcitabine) developed moderate leucopenia (2970/*μ*l) on postoperative day 3, and one patient under chronic anticoagulation developed an important intramural hematoma in the abdominal wall that was treated conservatively but required postoperative blood transfusion. In one patient, ascites leaked postoperatively through a trocar incision and necessitated a bedside skin suture. There were no procedure-related mortality and one hospital death (patient classified ASA IV, with 4500 ml ascites developed cardiopulmonary decompensation with fatal outcome on postoperative day 12)

### 3.4. Efficacy

**Figure 2:**
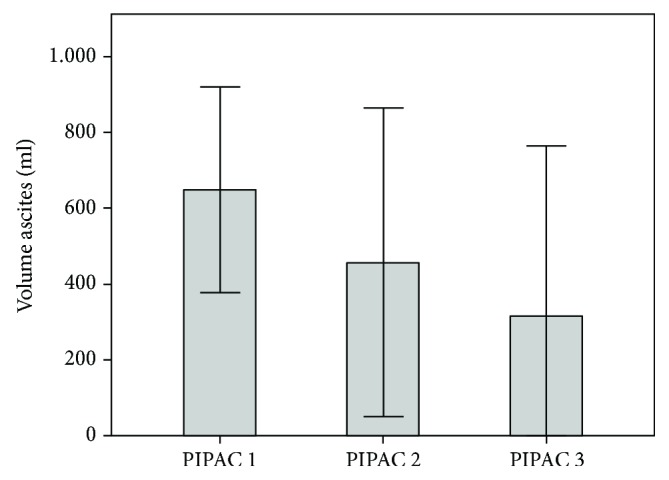
Ascites volume at PIPAC#1, PIPAC#2, and PIPAC#3. There is a significant decrease of ascites volume under therapy (ANOVA, *p* = 0.03).

39 patients underwent more than one PIPAC and were eligible for efficacy analysis. No radiological evaluation according to RECIST criteria was performed. Histological analysis was performed by an independent pathologist who could compare the current with previous biopsies. Peritoneal grading regression score (PRGS) could be calculated in 36 of these 39 patients. In 24 patients (67%), PRGS improved or remained unchanged at PIPAC#2, reflecting tumor regression or stable disease. In the remaining 12 patients, PRGS deteriorated under therapy. At PIPAC#2, 10/39 patients (26%) had a complete histological regression (PRGS = 1) in multiple peritoneal biopsies as well as in a local peritoneal peritonectomy sample. Ascites volume diminished between PIPAC#1 and PIPAC#3 (Figure 2); in patients with an initial ascites volume equal or superior to 300 ml, this difference was significant (ANOVA, *p* = 0.03).

### 3.5. Survival

**Figure 3:**
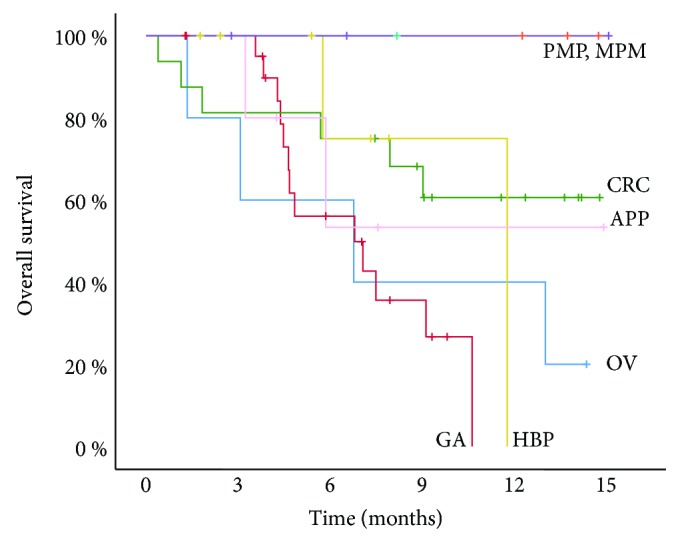
Overall survival probability (Kaplan-Meyer) of pretreated patients with pseudomyxoma peritonei (PMP), malignant peritoneal mesothelioma (MESO), and peritoneal metastasis of colorectal (CRC), appendiceal (APP), ovarian (OV), hepatobiliary-pancreatic (HBP), and gastric (GC) origins. Survival calculated from the date of first PIPAC.

The mean follow-up for all patients was 10.7 ± 4.4 months from PIPAC#1. At the end of the follow-up, 36/71 patients were alive, 19 were dead, and 6 were lost to follow-up. For all organs of origin together, the median survival from the first PIPAC was 11.8 months (95% CI: 7.45–16.2 months).  Figure 3 shows the overall survival from the first PIPAC depending on histology. The median survival from the first PIPAC was 6.8 months in ovarian cancer (median: 3–4^th^ line situation), 6.8 months also in gastric cancer (2^nd^ line situation), and 11.8 months in hepatobiliary-pancreatic tumors (3^rd^ line situation) and was not reached after 11.8 months for colorectal cancer (3-4^th^ line situation), pseudomyxoma peritonei (2-3^rd^ line situation), and mesothelioma (2^nd^ line situation).

## 4. Discussion

In the last few years, encouraging feasibility data regarding the application of PIPAC with low-dose cisplatin and doxorubicin or oxaliplatin in previously heavily pretreated patients with peritoneal metastasis have been published by several independent groups [17, 42]. Promising safety and efficacy results have been published in peritoneal metastasis of gastric [18, 19], ovarian [20, 21], colorectal [22], pancreatic [23, 24], and hepatobiliary [25] origins. Survey data on a total of 832 PIPAC procedures in 349 patients obtained from 9 centers have recently shown that the technique is well standardized with regard to indications, technical aspects, safety protocol, and treatment regimen so that results can be easily compared and even pooled between centers [43].

The results of this registry study on consecutive patients with advanced, pretreated peritoneal metastasis confirm that PIPAC is feasible, safe, and well-tolerated, which is in accordance with the above studies and with two systematic reviews [26, 44].

In the present cohort, the primary nonaccess rate was 8/71 patients = 11.2%. This figure is in line with the literature and confirms that nonaccess to the abdomen is a limitation of PIPAC, as it is the case in any staging laparoscopy after a previous abdominal surgery. However, the nonaccess rate appears to be influenced by patient selection, with a maximal risk of access failure in patients having received previous CRS and HIPEC. It is important to note that nonaccess is not harmful (as a rule). Thus, since there is no good alternative therapeutic option in the case of relapse, previous CRS and HIPEC are not an absolute contraindication for PIPAC.

A particular methodological problem of the available cohort studies on PIPAC is their heterogeneity and the difficulty of assessing objectively tumor response in peritoneal metastasis [45]. Contrast-enhanced CT-scan has a low sensitivity for small-volumetric peritoneal lesions [46], so that laparoscopic staging and biopsies are increasingly used not only for initial staging but also for response assessment on the basis of repeated biopsies [47].

In this cohort, after PIPAC therapy combined or not with systemic palliative chemotherapy, an objective histological response of peritoneal metastasis has been observed across various histologies in 2/3 of patients eligible for response assessment and a complete histological regression in one-fourth of them. This encouraging finding is the clinical translation of preclinical experiments documenting superior pharmacological properties of PIPAC, in particular, a higher drug tissue concentration, a deeper penetration of the drugs into tissues, and less systemic absorption [12]. This finding also confirms previous clinical data from phase II studies and patient cohorts showing histological response rates for therapy-resistant peritoneal metastasis of ovarian, colorectal, and gastric origins of 62–88, 71–86, and 70–100 percent, respectively (reviewed in 26). Thus, the results of this cohort fit well into the research map in this field and contribute to the body of evidence supporting the clinical efficacy of PIPAC as a palliative therapy of peritoneal metastasis.

Another promising finding in this study is significant ascites control already after the first cycle of intraperitoneal chemotherapy as PIPAC. The positive effect of PIPAC on ascites has already been reported in other studies [21, 48] and appears independent from the type of primary tumor and might contribute to the stabilization of the quality of life reported after PIPAC in several clinical studies [21, 45, 49] and patient cohorts [48, 50, 51].

Although the number of cases is limited, survival figures are encouraging. For example, the median survival of (heavily pretreated) patients with peritoneal metastasis of hepatobiliary-pancreatic origin was 11.8 months since first PIPAC. This survival time is unexpectedly long but confirms indeed a previous report with a median survival of 14 months (range 10–20) since the diagnosis of peritoneal metastasis [23]. The median survival of pretreated patients with peritoneal metastasis of gastric origin was 6.8 months, which is similar to our results (6.4 months) obtained within the framework of a phase II clinical trial in peritoneal metastasis of gastric origin in the salvage situation [49].

The registry data presented here are of exploratory nature, and caution is warranted in their interpretation. Extrapolation of these data to other patient cohorts or their use for individual therapeutic decisions is not permissible. However, the experimental finding remains that, in our patient cohort, PIPAC was safe and able to reverse platin resistance in the majority of patients, most of them having been heavily pretreated beforehand. Notably, objective tumor regression in the peritoneal tumor nodes was achieved with local administration of a dose of chemotherapy reduced by an order of magnitude (ten times) as compared to a systemic dose. This significant dose reduction contributes probably to the good tolerability of the procedure in the patients treated. In parallel to the high histological response rate, survival appears encouraging in this cohort and this is also in line with the superior survival statistics of four phase II trials examining the efficacy of PIPAC in advanced peritoneal metastasis [19, 21, 45, 49]. Several prospective randomized trials will now be required to confirm these encouraging results in each tumor entity with various potential treatment regimens.

## Figures and Tables

**Table 1:** Patient characteristics.

Variable	Value
Number of patients	71
Sex (M : F)	28 : 43
Age, years (±SD)	58 ± 13
Organ of origin	
(i) Gastric	26 (36.6%)
(ii) Colorectal	17 (23.9%)
(iii) Hepatobiliary-pancreatic	9 (12.7%)
(iv) Ovarian	6 (8.5%)
(v) Appendiceal	5 (7.0%)
(vi) PMP	4 (5.6%)
(vii) CUP, mesothelioma, yolk sac, prostate	3 (4.2%)
Extraperitoneal metastasis	
(i) Malignant pleural effusion	3 (4.2%)
(ii) Others	0
Peritoneal Cancer Index (PCI), mean ± SD	19.3 ± 12.5
Karnofsky Index before first PIPAC, mean ± SD	80.3 ± 14.7	
Previous surgery		
(i) CRS and HIPEC	10 (14.1%)	
(ii) Gastrectomy	11 (15.5%)	
(iii) Colectomy	11 (15.5%)	
(iv) Hysterectomy and adnexectomy	5 (7.0%)	
(v) Laparotomy	9 (12.7%)	
(vi) Laparoscopy	4 (5.6%)	
(vii) Other surgeries	14 (19.7%)	
(viii) None	7 (9.9%)	
Previous systemic chemotherapy		
(i) None	11 (15.5%)	
(ii) 1 line	18 (25.4%)	
(iii) 2 lines	17 (23.9%)	
(iv) 3 lines	10 (14.1%)	
(v) >3 lines	15 (21.1%)	
Simultaneous chemotherapy	42 (59.1%)	

**Table 2:** Adverse events.

Intraoperative			
Type of complication			
(i) Bowel injury	2^#^		
(ii) Lung aspiration	1		
(iii) Bleeding	1		
Total	4 (5.6%)		

Postoperative		Dindo-Clavien	CTCAE
Type of complication			
(i) Abdominal wall infiltration	2	1	N/A
(ii) Leucopenia	1	N/A	3
(iii) Ascites leakage	1	1	N/A
(iv) Nausea/vomiting	1	2	2
(v) Hematoma, transfusion	1	2	N/A
(vi) Hospital mortality	1^∗^	5	5
Total	7 (9.9%)		

^#^detected and repaired intraoperatively; ^∗^ASA IV patient, 4500 ml ascites, unrelated to procedure.

## Data Availability

The clinical data used to support the findings of this study are restricted by the Ethics Committee of Ruhr-University Bochum in order to protect patient privacy. Data are available from the institute for quality control in operative surgery at the University of Magdeburg, Germany (http://www.an-institut.de), for researchers who meet the criteria for access to confidential data.
